# Datasets linking ethnic perceptions to undergraduate students learning outcomes in a Nigerian Tertiary Institution

**DOI:** 10.1016/j.dib.2018.03.069

**Published:** 2018-03-22

**Authors:** Joke A. Badejo, Temitope M. John, David O. Omole, Emeka G. Ucheaga, Segun I. Popoola, Jonathan A. Odukoya, Priscilla O. Ajayi, Mary Aboyade, Aderemi A. Atayero

**Affiliations:** a Department of Electrical and Information Engineering, Covenant University, Ota, Ogun State, Nigeria; b Department of Civil Engineering, Covenant University, Ota, Ogun State, Nigeria; c Department of Banking and Finance, Covenant University, Ota, Ogun State, Nigeria; d Department of Psychology, Covenant University, Ota, Ogun State, Nigeria; e Center for Systems and Information Services, Covenant University, Ota, Ogun State, Nigeria; f Covenant University Data Analytics Cluster, Covenant University, Ota, Ogun State, Nigeria

**Keywords:** Learning analytics, Cultural impact, Ethnicity, Undergraduates, Education data mining, Smart campus, Nigerian university

## Abstract

This data article represents academic performances of undergraduate students in a select Nigerian Private Tertiary institution from 2008 to 2013. The 2413 dataset categorizes students with respect to their origins (ethnicity), pre-university admission scores and Cumulative Grade Point Averages earned at the end of their study at the university. We present a descriptive statistics showing mean, median, mode, maximum, minimum, range, standard deviation and variance in the performances of these students and a boxplot representation of the performances of these students with respect to their origins.

**Specifications Table**

**Table:** 

Subject area	*Education*
More specific subject area	*Learning Analytics*
Type of data	*Table, Figure, Graphs, and Spreadsheet*
How data was acquired	*The paper presents undergraduate students records retrieved from the department of records for a six-year study period between 2008 and 2013*
Data format	*Raw, analyzed*
Experimental factors	*Only students with complete records are retained in this study*
Experimental features	*Included in this paper are descriptive statistics, one-way ANOVA, box-plots*
Data source location	*The data was gathered from the department of students records at Covenant University, Ota, Nigeria (Latitude 6.67181*°*N, Longitude 3.1581*°*E)*
Data accessibility	*Data is attached in this article within the supplementary material section*

**Value of the data**

• The data provided will shed more light on students learning outcomes based on ethnicity; by providing sample data reflecting students’ academic performance based on ethnic groups.
• The data will provide empirical evidence confirming or refuting the impact of culture on students learning outcomes [1], [2].
• The data will be useful to advise education stakeholders in Nigeria and West Africa on the influence of ethnicity on students learning outcomes.

## 1 Data

**Fig. 1:**
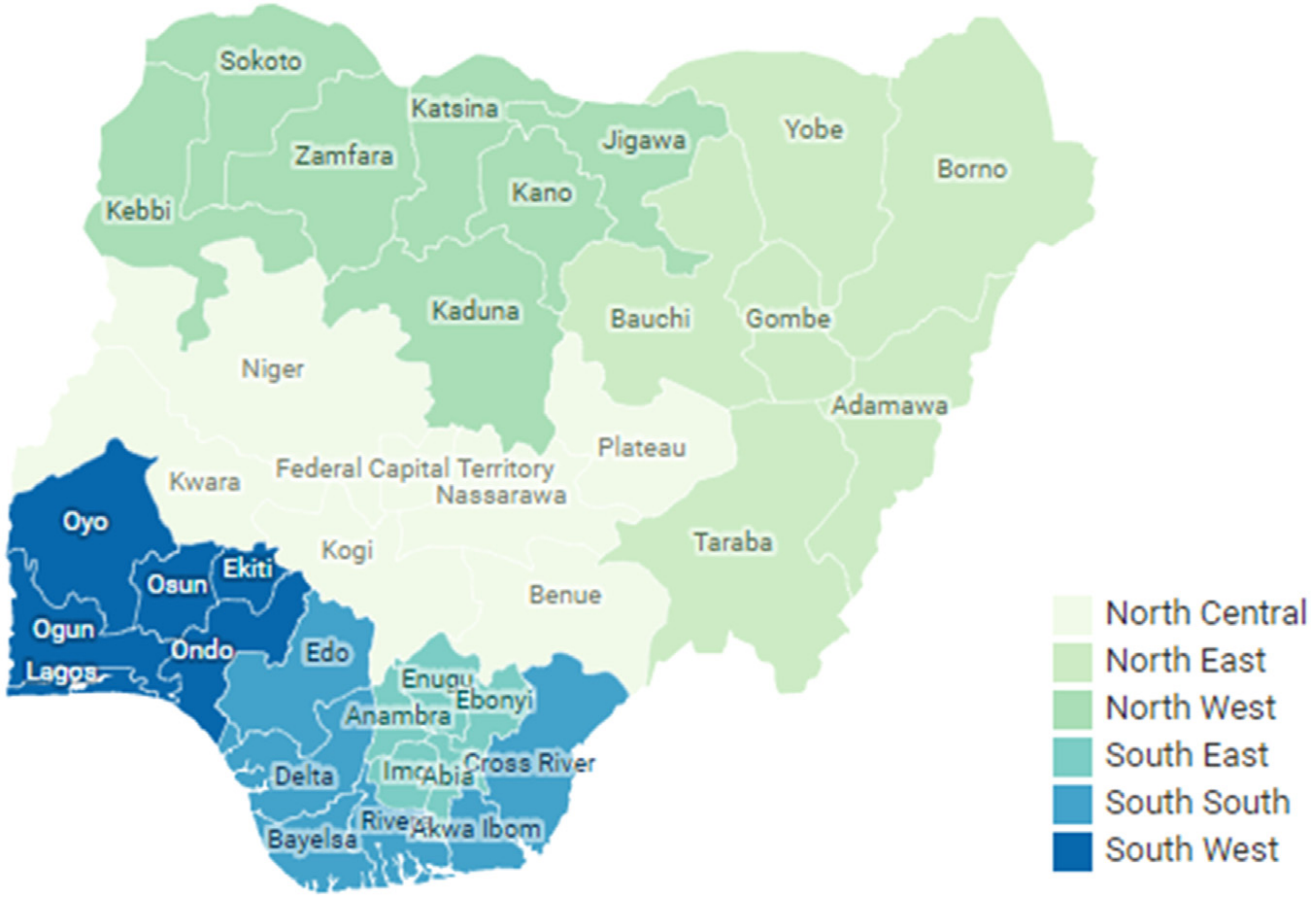
Map showing geopolitical zones in Nigeria.

The data presents a sample of students with respect to their origins from the six (6) geopolitical zones as identified by [3] in Nigeria; North-Central, North-East, North-West, South-East, South-South and South-West as shown in Fig. 1. These students have graduated from one of the four (4) colleges present in Covenant University, Ota, Nigeria. The students in this observation graduated between 2008 and 2013 (inclusive). The total sample size of this data is 2413, spread across 186, 40, 26, 409, 562, and 1190 students from North-Central, North-East, North-West, South-East, South-South and South-West respectively. The wide difference in the sample of students from some of the geopolitical zones is as a result of the degree of enrollment of students from those regions over the years. We present pre-university scores using the Joint Admission Matriculation Board (JAMB) examination scores [4] and the West Africa Examination Council (WAEC) examination scores [5]; these examinations are conducted by regulatory bodies in Nigeria and West Africa [6], [7]. University Scores are represented using the Cumulative Grade Point Average (CGPA).

## 2 Experimental design, materials, and methods

**Fig. 2:**
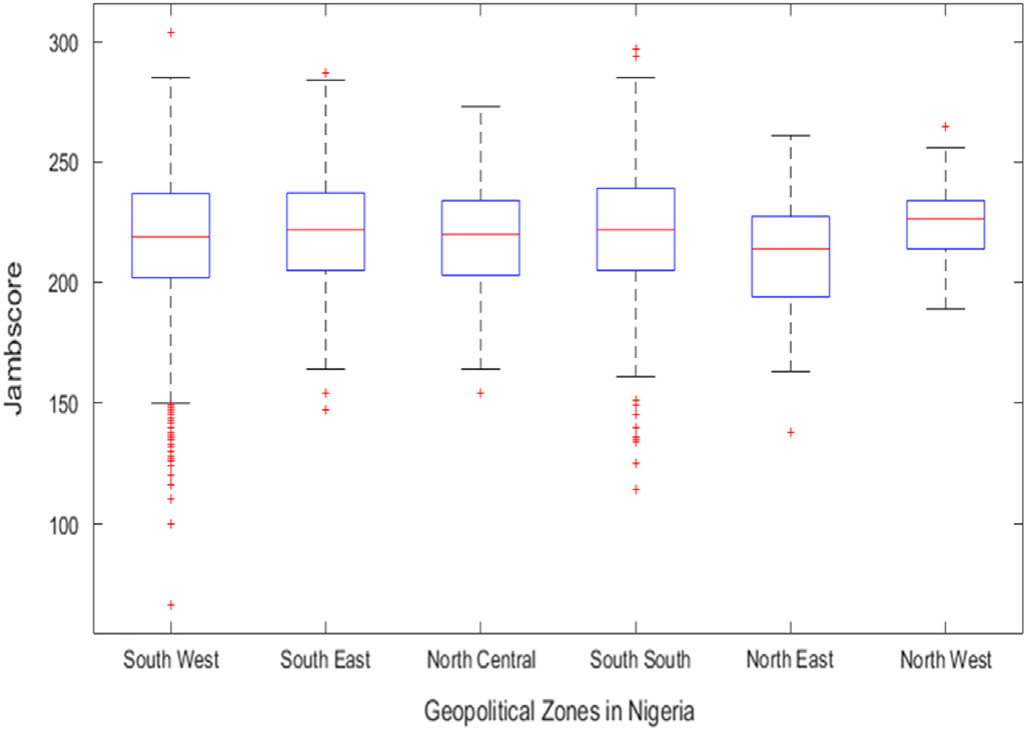
Boxplot of JAMB Score distribution by geopolitical zones in Nigeria (2008–2013).

**Fig. 3:**
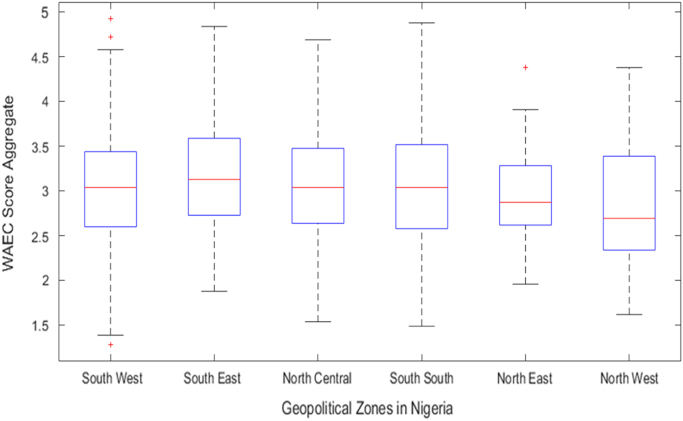
Boxplot of WAEC score aggregate distribution by geopolitical zones in Nigeria (2008–2013).

**Fig. 4:**
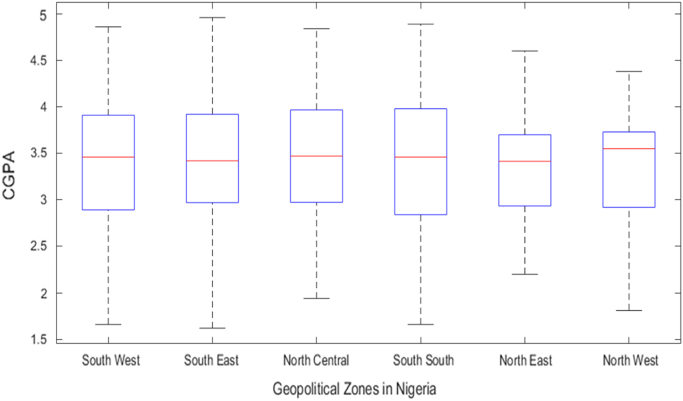
Boxplot of Cumulative Grade Point Average (CGPA) distribution by geopolitical zones in Nigeria (2008–2013).

The data was retrieved from the department of records and the Center for systems and information services at Covenant University. Table 1, Table 2, Table 3 show descriptive statistics of the performances of the students based on their pre-university and university scores. Fig. 2, Fig. 3, Fig. 4 show the boxplot distribution of JAMB, WAEC and CGPA scores for students based on the geopolitical zones representation from 2008 to 2013.

**Table 1:** Descriptive statistics of JAMB score of undergraduate students from 2008–2013.

**Geopolitical zone**	**Mean**	**Median**	**Mode**	**Maximum**	**Minimum**	**Range**	**Standard deviation**	**Variance**	Total N*
North-Central	219.30	220.00	220.00	273.00	154.00	119.00	22.42	502.80	186
North-East	209.05	214.00	182.00	261.00	138.00	123.00	24.67	608.72	40
North-West	224.54	226.50	198.00	265.00	189.00	76.00	18.47	341.06	26
South-East	221.29	222.00	209.00	287.00	147.00	140.00	24.14	582.80	409
South-South	221.22	222.00	224.00	297.00	114.00	183.00	27.20	740.06	562
South-West	217.62	219.00	231.00	304.00	66.00	238.00	28.00	783.87	1190

^*^Total *N* refers to population size of each sample.

**Table 2:** Descriptive statistics of WAEC score aggregate of undergraduate students from 2008–2013.

**Geopolitical zone**	**Mean**	**Median**	**Mode**	**Maximum**	**Minimum**	**Range**	**Standard deviation**	**Variance**	Total N*
North-Central	3.07	3.04	2.50	4.69	1.54	3.15	0.58	0.34	186
North-East	2.97	2.88	2.89	4.38	1.96	2.42	0.54	0.29	40
North-West	2.89	2.70	2.50	4.38	1.62	2.76	0.76	0.58	26
South-East	3.16	3.13	3.13	4.84	1.88	2.96	0.59	0.35	409
South-South	3.07	3.04	2.50	4.88	1.49	3.39	0.62	0.39	562
South-West	3.05	3.04	3.13	4.93	1.28	3.65	0.58	0.34	1190

^*^Total *N* refers to population size of each sample.

**Table 3:** Descriptive statistics of Cumulative Grade Point Average (CGPA) of undergraduate students from 2008–2013.

**Geopolitical zone**	**Mean**	**Median**	**Mode**	**Maximum**	**Minimum**	**Range**	**Standard deviation**	**Variance**	Total N*
North-Central	3.42	3.47	4.16	4.84	1.94	2.90	0.63	0.40	186
North-East	3.37	3.42	3.27	4.60	2.20	2.40	0.57	0.32	40
North-West	3.37	3.55	3.12	4.38	1.81	2.57	0.63	0.39	26
South-East	3.43	3.42	3.57	4.96	1.62	3.34	0.65	0.43	409
South-South	3.42	3.46	2.67	4.89	1.66	3.23	0.71	0.51	562
South-West	3.41	3.46	3.51	4.86	1.66	3.20	0.68	0.47	1190

^*^Total *N* refers to population size of each sample.
